# *Ureaplasma urealyticum* upregulates seminal fluid leukocytes and lowers human semen quality: a systematic review and meta-analysis

**DOI:** 10.1186/s12610-025-00262-5

**Published:** 2025-04-17

**Authors:** Omotola SeunFunmi Kukoyi, Victory Jesutoyosi Ashonibare, Cecilia Adedeji Adegbola, Tunmise Maryanne Akhigbe, Roland Eghoghosoa Akhigbe

**Affiliations:** 1 Functional Microbiome Group, Uniklinik Aachen (Universitätsklinikum Aachen), Aachen, Germany; 2 Reproductive Biology and Toxicology Research Laboratory, Oasis of Grace Hospital, Osogbo, Osun State Nigeria; 3 Cardiovascular Regenerative Medicine & Tissue Engineering 3D (CURE 3D) Lab, Department of Cardiovascular Surgery and Research Group for Experimental Surgery, Medical Faculty, Heinrich Heine University, Düsseldorf, Germany; 4 Department of Physiology, Ladoke Akintola University of Technology, Ogbomoso, Oyo State, Nigeria; 5 Department of Agronomy, Osun State University, Ejigbo Campus, Ejigbo, Osun State Nigeria

**Keywords:** Immunology, Infection, Male infertility, Semen, Testosterone, *Ureaplasma urealyticum*, Immunologie, Infection, Infertilité masculine, Sperme, Testostérone, Ureaplasma urealyticum

## Abstract

**Background:**

*Ureaplasma urealyticum* belongs to the class *Mollicutes* and causes non-gonococcal urethritis, an inflammation of the urethra that is linked with impaired semen quality. However, some reports are contradictory, and the reported effect of *U. urealyticum* on specific sperm variables is not consistent. Thus, this study synthesized findings from published primary data and provides a robust and reliable inference on the impact and associated mechanisms of *U. urealyticum* on sperm quality.

**Methods:**

A systematic search was conducted until 31st May, 2024, on Cochrane, Google Scholar, and Pubmed. The Population, Exposure, Comparator/Comparison, Outcomes, and Study Design (PECOS) model was adopted. The populations were male in their reproductive ages who were infected with *Ureaplasma urealyticum* and confirmed positive versus the control who were age-matched non-infected or treated, while the outcomes included conventional semen parameters, seminal fluid leucocyte count, and sperm interleukin-6 (IL-6) concentrations, and the studies were either cross-sectional or longitudinal.

**Results:**

When compared with the control, quantitative analysis demonstrated that *U. urealyticum* significantly reduced ejaculate volume (SMD 0.33 [95% CI: 0.15, 0.52] *p* = 0.0004), sperm concentration (SMD 0.47 [95% CI: 0.31, 0.64] *p* < 0.00001), total sperm motility (SMD 0.73 [95% CI: 0.43, 1.02] *p* < 0.00001), total motile sperm count (SMD 0.21 [95% CI: 0.17, 0.26] *p* < 0.00001), normal sperm morphology (SMD 0.88 [95% CI: 0.42, 1.35] *p* = 0.0002), but increased seminal fluid leukocyte count (SMD -0.82 [95% CI: -1.61, -0.02] *p* = 0.04). In addition, qualitative analysis revealed that *U. urealyticum-*positive subjects had significantly higher levels of IL-1β, IL-6, IL-8, TNF-α, peroxidase, *leukocytes*, neutrophils, CD4 + T cells, and CD8 + T cells in the seminal fluid when compared with the control. Furthermore, higher sperm DNA fragmentation and apoptotic sperm cells were observed in *U. urealyticum-*positive subjects when compared to the control.

**Conclusions:**

These findings revealed that *U. urealyticum* lowers semen quality via the upregulation of seminal fluid leukocytes, elastase, pro-inflammatory cytokines, and DNA fragmentation. However, further studies are required to elucidate the mechanisms underlying the association between *U. urealyticum* and semen quality decline and to develop effective therapies for this condition.

**Supplementary Information:**

The online version contains supplementary material available at 10.1186/s12610-025-00262-5.

## Résumé

**Contexte:**

*Ureaplasma urealyticum* appartient à la classe des *Mollicutes* et provoque une urétrite non gonococcique, une inflammation de l’urètre, qui est liée à une altération de la qualité du sperme. Cependant, certains rapports sont contradictoires, et l’effet rapporté de *U. urealyticum* sur les paramètres spécifiques des spermatozoïdes n’est pas cohérent. La présente étude a synthétisé les résultats des données primaires publiées, et fournit une inférence robuste et fiable sur l’impact et les mécanismes associés d’*U. urealyticum *sur la qualité du sperme. Une recherche systématique a été menée jusqu’au 31 mai 2024 sur Cochrane, Google Scholar et PubMed. Le modèle PECOS (Population, Exposure, Comparator/Comparison, Outcomes, and Study Design) a été adopté. Les populations étaient des hommes en âge de procréer et infectés par *U urealyticum,* et dont les résultats positifs étaient confirmés par rapport aux témoins appariés selon l’âge, non infectés ou traités ; les résultats comprenaient les paramètres conventionnels du sperme, le nombre de leucocytes dans le liquide séminal et les concentrations d’interleukine-6 des spermatozoïdes (IL-6).

**Résultats:**

Par rapport aux témoins, l’analyse quantitative a montré que *U. urealyticum *réduisait significativement le volume d’éjaculat (Différence de Moyenne Standardisée DMS 0,33 [IC à 95 % : 0,15, 0,52] p = 0,0004), la concentration de spermatozoïdes (DMS 0,47 [IC à 95 % : 0,31, 0,64] p< 0,00001), la motilité totale des spermatozoïdes (DMS 0,73 [IC à 95 % : 0,43, 1,02] p< 0,00001), le nombre total de spermatozoïdes mobiles (DMS 0,21 [IC à 95 % : 0,17, 0,26] p< 0,00001), les spermatozoïdes de morphologie normale (DMS 0,88 [IC à 95 % : 0,42, 1,35] p = 0,00002), mais augmentait la numération leucocytaire du liquide séminal (DMS -0,82 [IC à 95 % : -1,61, -0,02] p = 0,04). De plus, l’analyse qualitative a révélé que les sujets positifs à *U. urealyticum* présentaient des taux significativement plus élevés d’IL-1β, d’IL-6, d’IL-8, de TNF-α, de peroxydase, de leucocytes, de neutrophiles, de lymphocytes T CD4+ et de lymphocytes T CD8+ dans le liquide séminal par rapport aux témoins. De plus, des taux plus élevés de fragmentation de l’ADN des spermatozoïdes et de spermatozoïdes apoptotiques ont été observées chez les sujets positifs à *U. urealyticum* par rapport aux témoins.

**Conclusions:**

Ces résultats ont révélé que *U. urealyticum* abaisse la qualité du sperme via la régulation positive des leucocytes du liquide séminal, de l’élastase, des cytokines pro-inflammatoires et de la fragmentation de l’ADN. Cependant, d’autres études sont nécessaires pour élucider les mécanismes sous-jacents à l’association entre *U. urealyticum* et le déclin de la qualité du sperme, ainsi que pour développer des traitements efficaces pour cette maladie.

## Introduction

Infertility is the failure to achieve conception after a year of adequate unprotected sexual activity [1]. If this is due to a male factor, it is referred to as male infertility. About 30–50% infertility cases are caused by male factors, either solely or in combination with female factors [2]. Although there are emerging causes, predisposing factors of male infertility are classified as pre-testicular, testicular, and post-testicular factors. Post-testicular causes include *vas deferens* blockage, ejaculatory duct obstruction, retrograde ejaculation and cystic fibrosis, while testicular factors include testicular torsion/detorsion, varicocele, cryptorchidism, testicular cancer, heat stress, Orchitis, spermatocele, genetic disorders, and pre-testicular causes include endocrinopathies, smoking, use of illicit drugs, psychoemotional stress, thyroid disorders, exposure to environmental toxicants such as heavy metals, and infections [2–6]. These infections may be viral [7, 8], fungal [9], or bacterial [10]. One of the most prevalent bacterial causes of male infertility is *Ureaplasma urealyticum* [11, 12]*.*

*Ureaplasma urealyticum* belongs to class *Mollicutes*, commonly knowns as Mycoplasma. Although commensal in the human urogenital tracts, they could be opportunistic, causing variety of reproductive diseases and disorders. Ureaplasma spp hydrolyze urea and release ammonia, they produce IgA protease, phospholipases A and C as well as hydrogen peroxide. *Ureaplasma urealyticum* causes non-gonococcal urethritis (NGU) [13]; an inflammation of the urethra without the presence of *Neisseria gonorrhoeae*. The symptoms of NGU include penile discharge, dysuria, and irritation around the urethra; these are mainly linked to a decrease in male fertility due to their effects on sperm quality [14]. *Ureaplasma urealyticum* spreads through several routes, such as direct sexual contact between partners, vertical transmission from mother to child, hospital-contracted infections from transplanted tissues, or unprotected anal, vaginal, or oral sex with an infected individual [15]. *Ureaplasma urealyticum* has been linked with central nervous system infections, bronchopulmonary dysplasia, sexually transmitted reactive arthritis (Reiter's syndrome), chronic prostatitis, epididymitis, and pregnancy complications [16].

*U. urealyticum* infects sperm cells by attaching and integrating itself to the head of the sperm cells, thus leading to a reduction in sperm count, concentration, viability, and motility [17, 18]. *U. urealyticum* also negatively impacts semen pH and sperm morphology [19]. *U. urealyticum* triggers inflammation [11] and enhances reactive oxygen species (ROS) generation [20], leading to damage to the sperm membrane, proteins and deoxyribonucleic acid (DNA), thus DNA fragmentation. In addition, *U. urealyticum* activates the immune system and promotes the production of antisperm antibodies [21] that may bind to spermatozoa and impair their motility and oocyte-penetration ability, thus reducing male fertility. *U. urealyticum* may also alter the chemico-biological makeup of the semen by altering the seminal fluid pH [17, 18], making the environment hostile to sperm survival and motility.

Despite several reports on the negative impact of *U. urealyticum* on sperm quality and male fertility, some reports are conflicting and the reported effect of *U. urealyticum* on specific sperm variable is not consistent. Thus, the present study synthesized findings from multiples primary data and provides a more robust and reliable conclusion on the impact and associated mechanisms of *U. urealyticum* on sperm quality.

## Materials and methods

### Protocol and inclusion criteria

The present study was registered on PROSPERO (CRD42024534950). This study adhered to the “Preferred Reporting Items for Systematic Reviews and Meta-analyses (PRISMA)” strategy. The Population, Exposure, Comparator/Comparison, Outcomes, and Study design (PECOS) model was adopted and eligible studies published until May, 2024 were included. The studied populations were male in their reproductive ages who were infected with *Ureaplasma urealyticum* and confirmed positive. The outcomes included ejaculate volume, seminal fluid pH, sperm concentration, total motility, progressive motility, total motile sperm count, sperm vitality (viability), sperm morphology, and seminal fluid leucocyte count and interleukin- 6 (IL- 6) concentrations. The studies were either cross-sectional or longitudinal. Studies without appropriate control groups, studies in female, in vitro studies, review articles, commentaries, perspectives, letters to editor, editorials, preprint, conference abstract, degree thesis, and retracted papers were excluded. There was no restriction on language or country.

Search strategy, assessment of the quality of the eligible studies, and data collection.

Systematic searches were conducted on Cochrane, Google Scholar, and Pubmed using these Boolean strings ("Ureaplasma urealyticum"OR"urealyticum"OR"ureaplasma") AND ("sperm"OR"semen"OR"sperm count"OR"sperm concentration"OR"sperm motility"OR"semen volume"OR"ejaculate volume"OR"semen pH"OR"semen leucocyte"OR"semen WBC"OR"sperm viability"OR"sperm vitality").

Abstracts and full text of articles were collected by all authors, and the eligible studies were assessed for the quality of evidence (QoE), risk of bias (RoB), and certainty of evidence (CoE) by OSK, VJA, CAA, and TMA. Disputes were resolved by REA. The QoE of eligible papers was evaluated by the ErasmusAGE quality score for systematic reviews, which assigns a number between 0 and 2 to five different domains, and a possible maximum score of 10 [22], while the “Office of Health Assessment and Translation (OHAT)” methodology was used to evaluate the RoB [23] and Using the “Grading of Recommendations Assessment, Development and Evaluation (GRADE) Working Group” standards as a guide, the “OHAT approach for systematic review and evidence integration for literature-based health assessment was used to assess the certainty of the evidence” [24, 25].

The data collected from the eligible studies include: last name of the principal investigator, publication date, study design, town and country of study origin, method of *Ureaplasma urealyticum* diagnosis, sample size, ages of patients, and measured outcomes of interest. The outcomes of interest were collected as mean and standard deviation, but when the variables were presented in other forms, the mean and standard deviation were derived from the provided data. Web Plot Digitizer was employed to derive the quantitative data in cases where graphs were used.

Quantitative and qualitative analysis.

Where sufficient data were collected, meta-analyses were conducted using Review Manager (version 5.4.1). The standardized mean difference (SMD) at 95% confidence intervals (CIs) was calculated. When *p*-value < 0.1 or I^2^ > 50% which indicates the presence of significant heterogeneity, a random-effect model was used; however, when *p*-value > 0.1 or I^2^ < 50% which suggests the absence of significant heterogeneity, a fixed-effect model was used. Subgroup analyses were performed per study design (cross-sectional studies and longitudinal studies) to evaluate the impact of *Ureaplasma urealyticum* infection in patients versus control, and before versus after *Ureaplasma urealyticum* treatment on various endpoints respectively. The controls were age-matched uninfected subjects, but in longitudinal studies, the control refers to both uninfected subjects and the treated state as indicated in the figures. Subgroup analyses were also performed based on the different diagnostic techniques [Polymerase Chain Reaction (PCR), culture or rapid diagnostic tests (RDT)]. To assess the possible sources of heterogeneity, sensitivity analysis was conducted by excluding the studies with the largest weight, low QoE (< 5), high RoB (< 4), and low CoE. Publication bias was visually determined using the funnel’s plots.

Where there were limited study and data collected were not sufficient for quantitative analysis, qualitative analyses were done.

## Results

### Study identification, screening, and inclusion

**Fig. 1:**
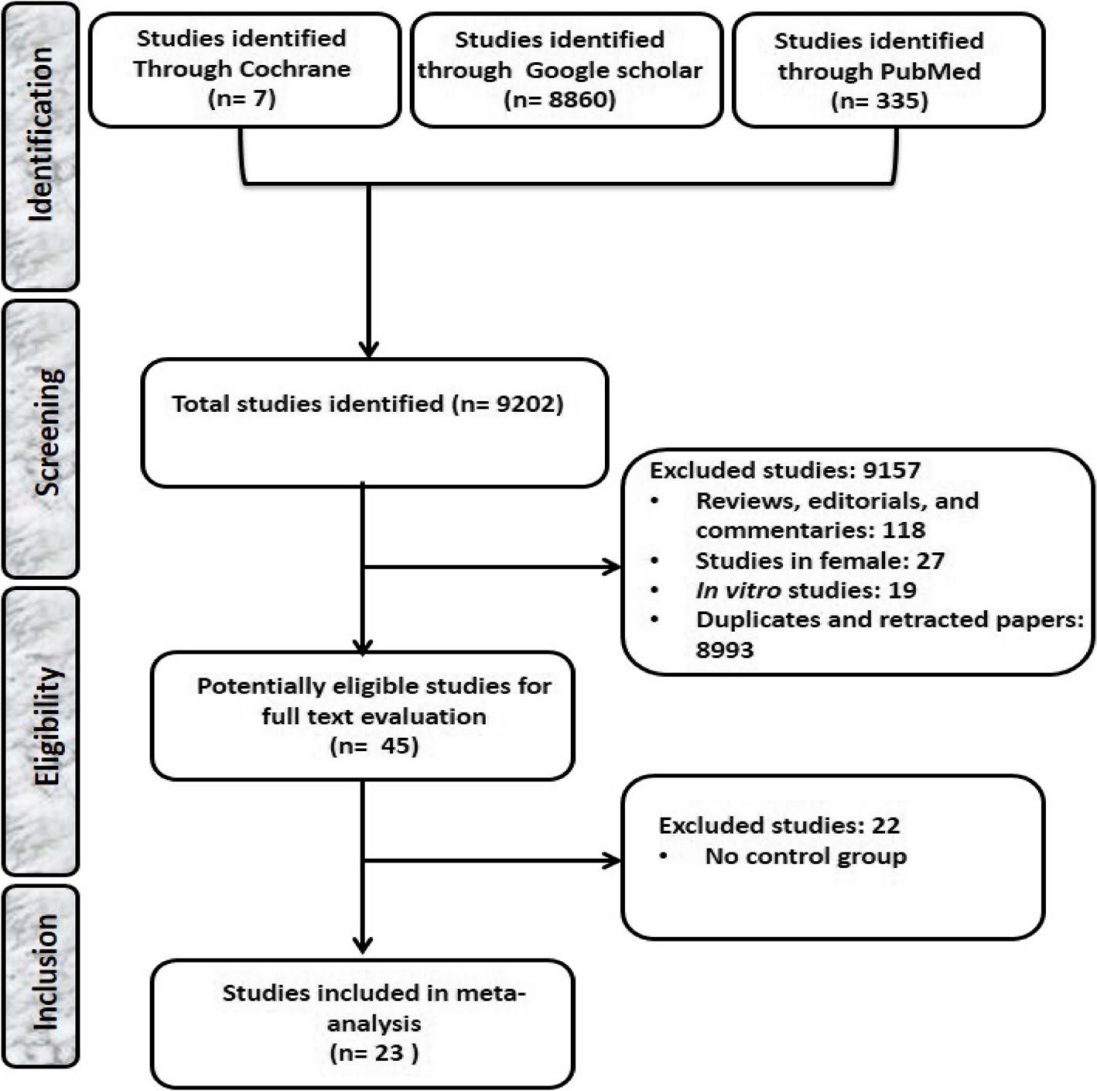
PRISMA flowchart for the strategic identification, screening, and inclusion of eligible studies

A total of 9202 published articles were identified from the various databases. After the exclusion of review articles, editorials, commentaries, studies in female, in vitro studies, duplicates and retracted papers, 45 articles were potentially eligible for full text evaluation. Out of this, 22 were excluded for lack of appropriate comparison, leaving 23 articles [11, 12, 17, 17, 18, 26–44] as eligible for inclusion in the study (Fig. 1).

The eligible papers were published between 2003 and 2024, and they were from diverse geographical location. Other details of the included studies such as the diagnostic techniques, sample size, and age distribution of subjects are presented in Table 1.

**Table 1:** Characteristics of the eligible studies included in the meta-analysis

References	Study design	Town/Country	Diagnosis of U.U	Examined population	Age (years)	Outcomes/variables measured
Aghazarian et al., 2024 [26]	Cross-sectional	Hietzing, Austria	Culture based method	UU (n = 27), two ≥ 2 pathogenic species (n = 28), Gardnerella Vaginalis (n = 15), gram-positive cocci and bacilli (n = 15), gram-negative (n = 10) and Chlamydia trachomatis (n = 2). One control group (n = 20) and one leukocytospermic group (n = 10)		Sperm concentration, progressive motility, total motility, sperm count, vitality, normal morphology, leukocyte, IL- 6
Al-Daghistani & Abdel-Dayem, 2010 [27]	Cross-sectional	Jordan	PCR	infertile patients: 99 Fertile control: 21		Progressive motility, sperm count
AL-SWEIH, et al., 2012 [28]	Cross-sectional	Kuwait	PCR	127 infertile and 188 fertile men		Seminal volume, pH, sperm concentration, vitality, total progressive motility category [a + b], rapid progressive motility category [a], morphology (normal forms), and white blood cell (WBC) counts
Andrade-Rocha, 2003 [29]	Cross-sectional	?? Brazil	Culture based	234	17 to 60 yrs	Sperm count, viability, progressive motility, total motility, normal morphology
Bai et al., 2021 [30]	Cross-sectional	China	Flow-through hybridization technique using the STD (sexually trans- mitted disease) 6 Geno Array Diagnostic kit	LCS: 88 Non-LCS:107	32.4 ± 6.9 and 31.6 ± 5.8 years	Semen volume, concentration, progressive motility, total motility, normal morphology, leukocytes, AsAs
Gdoura et al., 2007 [31]	Cross-sectional	Tunisia	PCR	Pos: 18 Neg: 102	26- 58	Volume, pH, concentration, total progressive motility, rapid progressive motility, morphology, leukocyte count
Golshani et al., 2007 [32]	Cross-sectional	Iran	PCR	Total 200 CT 18 MH 22 UU 6 MH, CT 14 MH, UU 2 UU, CT 2 CT, MH, UU 2 Neg 134	20- 50	Volume, pH, concentration, rapid progressive motility, slow progressive motility, non-progressive Motility, non-motile, abnormal morphology, leukocytospermia
Karthikeyan et al., 2021 [33]	Cross-sectional	India	PCR	PCR + : 8 PCR-: 40	32–38.75	Volume, concentration, total motility, progressive motility, immotility, normal forms
Huang et al., 2015 [34]	Cross-sectional	China	Mycoplasma IST (rapid detection)	19,098 semen specimens from infertile 3368 semen specimens’ control	23–44 years patient 20–35 years control	Volume, pH, sperm concentration, PR, total motility, normal morphology, sperm count/TMC
Le et al., 2020 [35]	Cross-sectional	Vietnam	PCR	Pos: 61 Neg: 319	20- 45	pH, volume, concentration, sperm PR Motility, normal morphology, sperm vitality
Liu et al., 2014 [36]	Cross-sectional	?? China	Rapid detection	621 infertile and 615 fertile men		Volume, sperm concentration, total motility, PR, vitality, sperm count
Liu et al., 2022a [17]	Cross-sectional and longitudinal	Guangdong, China	Culture-based method	Patient:104 Control: 236	21 ~ 46 years	Semen volume, pH, concentration, immotility, VCL: curvilinear velocity; VSL: straight-line (rectilinear) velocity; VAP: average path velocity
Liu et al., 2022b [18]	Cross-sectional and longitudinal	Guangdong, China	Culture-based method	198 normal semen samples (control group) and 198 UU-infected semen samples	20 to 45 years	men volume, pH, viscosity, liquefaction time, sperm concentration, and sperm motility [progressive motility (PR), Seminal PMN elastase
Moretti et al., 2009 [37]	Cross-sectional	?? Italy	Transmission electron microscopy, culture	Control: 20 E. faecalis (70) E. coli (46) S. agalactiae (33) U. urealyticum (28) S.epidermidis (24) S. anginosus (21) M. morganii (8)	Control 22- 35	Volume, sperm number, motility, fertility index, apoptosis, necrosis, immaturity
Paira et al., 2023 [11]	Cross-sectional	?? Argentina	PCR	212 males	18 to 50 yr	IL- 8, TNF, IL- 1β, IL- 6, IFNγ, IL- 10, and IL- 17 A concentration
Peerayeh et al., 2008 [38]	Cross-sectional	Iran	PCR	Varicocele + 81 Varicocele – 65 Healthy 100	21 − 50 Control (20- 40)	Volume, motility, sperm count, morphology
Pendas et al., 2013 [39]	Cross-sectional	La Havana	Culture-based	140 men	20- 45 years	Volume, sperm concentration, motility, morphology, leukocyte count
Qing et al., 2017 [40]	Cross-sectional	China	PCR	Uninfected 957 CT C. trachomatis only 27 MG M. genitalium only 51 NG N. gonorrhoeae only 6 UU U. urealyticum only 1418 Mixed infection 148		Concentration, volume, PR, normal morphology, DFI, HDS
Xianchun et al., 2023 [12]	Cross-sectional	China	PCR	U. urealyticum infection (n = 328), (ii) non-U. urealyticum infection n = 377), and (iii) normal volumeunteers n = 359)	22—30	Semen volume, density, activation rate, forward movement, survival rate, normal sperm morphology, pH; liquefaction time, density, non-forward, in active, VAP, VCL, ALH,BCF, VSL, motility, WOB, LIN, STR, viscosity
Zeighami et al., 2007 [41]	Cross-sectional	?? Iran	PCR	Infertile 100 Control 100	??	Volume, motility, sperm count, morphology,
Zeighami et al., 2009 [42]	Cross-sectional	Iran	PCR	Infertile 100 Control 100	Control 20–40 Patient 21–50	Volume, motility, sperm count, morphology,
Zhang et al., 2020 [43]	Cross-sectional and longitudinal	China		Non- leukocytospermia group 1235 Leukocytospermia group 295 UU-negative leukocytospermia group 200 UU-positive leukocytospermia group 95	29–37 yrs	Semen volume, pH, concentration, PR, total motility, normal morphology
Zinzendorf et al., 2008 [44]	Cross-sectional	Côte d’Ivoire	Culture based	1 058 semen samples	23–51 years	Volume, viscosity, liquefaction, pH, motility, morphology, concentration, leuckocyte count

*UU Ureaplasma urealyticum*, *PCR* Polymerase chain reaction, *PR* Progressive motility, *VAP* average path velocity, *VCL* Curvilinear velocity, *ALH* Amplitude of lateral head displacement, *BCF* Beat cross frequency, *VSL* Straight-line velocity, *WOB* Wobble, *LIN* Linearity, *STR* Straightness, *DFI* DNA fragmentation index, *HDS* High DNA stainability, *IL* Interleukin, *TNF* Tumour necrosis factor, *IFNγ* Gamma interferon, *PMN* Polymorphonuclear leukocytes

### Assessment of the QoE, RoB, and CoE

All the eligible studies [11, 12, 17, 18, 26–44] had QoE above 5, showing good QoE (Table 2). However, four of the studies [27, 29, 33, 39] had moderate (6/9) RoB while others [11, 12, 17, 18, 26, 28, 30–32, 34–38, 40–44] had low (> 6/9) RoB (Table 3). In addition, three of the eligible studies [38, 39, 43] had moderate CoE, while others [11, 12, 17, 18, 26–37, 40–42, 44] had high CoE (Table 4).

**Table 2:** Assessment of the quality of evidence of the eligible studies

Study	Study design	Study size	Method of measuring exposure	Method of measuring outcome	Analysis with adjustment	Total
Aghazarian et al., 2024 [26]	1	2	1	2	1	7/10
Al-Daghistani & Abdel-Dayem, 2010 [27]	1	1	2	2	1	7/10
AL-SWEIH, et al., 2012 [28]	1	2	2	2	2	9/10
Andrade-Rocha, 2003 [29]	1	1	1	1	1	5/10
Bai et al., 2021 [30]	1	2	1	1	2	7/10
Gdoura et al., 2007 [31]	1	1	2	1	1	6/10
Golshani et al., 2007 [32]	1	2	2	1	2	8/10
Huang et al., 2015 [33]	1	2	1	1	2	7/10
Karthikeyan et al., 2021 [34]	1	0	2	2	2	7/10
Le et al., 2020 [35]	1	2	2	1	2	7/10
Liu et al., 2014 [36]	1	2	1	2	1	7/10
Liu et al., 2022a [17]	2	1	1	2	2	8/10
Liu et al., 2022b [18]	1	2	1	2	1	7/10
Moretti et al., 2009 [37]	1	2	2	2	2	9/10
Paira et al., 2023 [11]	1	2	2	2	2	9/10
Peerayeh et al., 2008 [38]	1	2	2	1	1	7/10
Pendas et al., 2013 [39]	1	1	1	2	1	6/10
Qing et al., 2017 [40]	1	2	2	1	2	8/10
Xianchun et al., 2023 [12]	1	2	2	2	2	9/10
Zeighami et al., 2007 [41]	1	2	2	1	1	7/10
Zeighami et al., 2009 [42]	1	2	2	1	1	7/10
Zhang et al., 2020 [43]	1	2	1	1	2	7/10
Zinzendorf et al., 2008 [44]	1	2	1	1	2	7/10

**Table 3:** Risk of bias assessment of the eligible studies

Study	Selection of exposed cohort	Selection of non-exposed cohort	Assessment of exposure	Demonstration of outcome	Comparability (basics)	Comparability (others)	Assessment outcome	Length of follow-up	Adequacy of follow-up	Total (star)
Aghazarian et al., 2024 [26]	1	1	1	1	1	1	1	0	0	7
Al-Daghistani & Abdel-Dayem, 2010 [27]	1	1	1	1	1	0	1	0	0	6
AL-SWEIH, et al., 2012 [28]	1	1	1	1	1	1	1	0	0	7
Andrade-Rocha, 2003 [29]	1	1	1	1	1	0	1	0	0	6
Bai et al., 2021 [30]	1	1	1	1	1	1	1	0	0	7
Gdoura et al., 2007 [31]	1	1	1	1	1	1	1	0	0	7
Gdoura et al., 2007 [32]	1	1	1	1	1	1	1	0	0	7
Huang et al., 2015 [33]	1	1	1	1	1	1	0	0	0	6
Karthikeyan et al., 2021 [34]	1	1	1	1	1	1	1	0	0	7
Le et al., 2020 [35]	1	1	1	1	1	1	1	0	0	7
Liu et al., 2014 [36]	1	1	1	1	1	1	1	0	0	7
Liu et al., 2022a [17]	1	1	1	1	1	1	1	1	1	9
Liu et al., 2022b [18]	1	1	1	1	1	1	1	0	0	9
Moretti et al., 2009 [37]	1	1	1	1	1	1	1	0	0	7
Paira et al., 2023 [11]	1	1	1	1	1	1	1	0	0	7
Peerayeh et al., 2008 [38]	1	1	1	1	1	1	1	0	0	7
Pendas et al., 2013 [39]	1	1	1	1	1	0	1	0	0	6
Qing et al., 2017 [40]	1	1	1	1	1	1	1	0	0	7
Xianchun et al., 2023 [12]	1	1	1	1	1	1	1	0	0	7
Zeighami et al., 2007 [41]	1	1	1	1	1	1	1	0	0	7
Zeighami et al., 2009 [42]	1	1	1	1	1	1	1	0	0	7
Zhang et al., 2020 [43]	1	1	1	1	1	1	1	0	0	7
Zinzendorf et al., 2008 [44]	1	1	1	1	1	1	1	0	0	7

**Table 4:** Assessment of the certainty of evidence of the eligible studies

Study	Initial rating	Downgrading?	Upgrading?	Confidence in body of evidence
Aghazarian et al., 2024 [26]	High	No	No	High
Al-Daghistani & Abdel-Dayem, 2010 [27]	High	No	No	High
AL-SWEIH, et al., 2012 [28]	High	No	Yes, 1	High
Andrade-Rocha, 2003 [29]	High	No	No	High
Bai et al., 2021 [30]	High	No	No	High
Gdoura et al., 2007 [31]	High	No	No	High
Golshani et al., 2007 [32]	High	No	No	High
Huang et al., 2015 [33]	Medium	No	Yes, 1	High
Karthikeyan et al., 2021 [34]	High	No	No	High
Le et al., 2020 [35]	High	No	Yes, 1	High
Liu et al., 2014 [36]	High	No	Yes, 1	High
Liu et al., 2022a [17]	High	No	Yes, 1	High
Liu et al., 2022b [18]	High	No	No	High
Moretti et al., 2009 [37]	Moderate	No	Yes, 1	High
Paira et al., 2023 [11]	High	No	No	High
Peerayeh et al., 2008 [38]	High	Yes, 1	No	Moderate
Pendas et al., 2013 [39]	Moderate	No	No	Moderate
Qing et al., 2017 [40]	High	No	Yes, 1	High
Xianchun et al., 2023 [12]	High	No	Yes, 1	High
Zeighami et al., 2007 [41]	High	No	No	High
Zeighami et al., 2009 [42]	High	No	No	High
Zhang et al., 2020 [43]	High	Yes, 1	No	Moderate
Zinzendorf et al., 2008 [44]	High	No	Yes, 1	High

### Quantitative analysis

#### Ejaculate volume

**Fig. 2:**
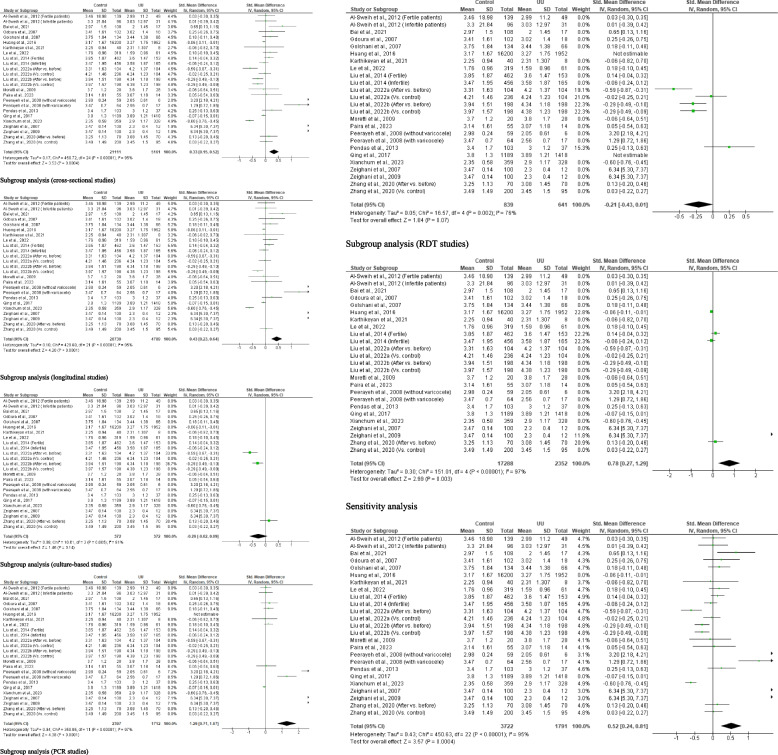
Effect of *ureaplasma urealyticum* on human ejaculate volume. UU: *Ureaplasma urealyticum,* SD: Standard deviation, CI: confidence interval. Meta-analysis was performed using a random-effect model. Data are shown as standardized mean difference and Confidence interval (CI)

A total of 25 studies from 19 published articles consisting of 21, 111 controls and 5, 161 *U. urealyticum*-infected subjects were included to determine the effect of *U. urealyticum* on ejaculate volume. It was observed that *U. urealyticum* significantly reduced ejaculate volume when compared with the control (SMD 0.33 [95% CI: 0.15, 0.52] *p* = 0.0004). More so, there was a significant heterogeneity (I^2^ = 95%; *X*^2^ *p* < 0.00001). Publication bias was also observed (S Fig. 1). The subgroup analysis of the cross-sectional studies revealed a significant reduction in the ejaculate volume of in *U. urealyticum-*infected subjects when compared with *U. urealyticum-*negative subjects (SMD 0.43 [95% CI: 0.23, 0.64] *p* < 0.0001). Also, a significant heterogeneity was observed (I^2^ = 95%; *X*^2^ *p* < 0.00001). However, subgroup analysis of the longitudinal studies showed that *U. urealyticum* did not significantly alter ejaculate volume (SMD − 0.26 [95% CI: 0.62, 0.09] *p* = 0.14) and a significant heterogeneity was also observed (I^2^ = 81%; *X*^2^ *p* = 0.005). More so, the subgroup analysis of the culture-based studies showed that *U. urealyticum* significantly reduced ejaculate volume (SMD 1.29 [95% CI: 0.71, 1.87] *p* < 0.0001) but a significant heterogeneity was also observed (I^2^ = 97%; *X*^2^ *p* < 0.00001), while the subgroup analysis of the PCR-based studies showed that *U. urealyticum* did not alter ejaculate volume (SMD − 0.21 [95% CI: − 0.43, 0.01] *p* = 0.07) and a significant heterogeneity was also observed (I^2^ = 76%; *X*^2^ *p* = 0.002). The subgroup analysis of the RDT-based studies demonstrated a significant reduction in ejaculate volume in patients infected with *U. urealyticum* when compared with the control (SMD 0.78 [95% CI: 0.27, 1.29] *p* = 0.003) but a significant heterogeneity was also observed (I^2^ = 97%; *X*^2^ *p* < 0.00001). Nonetheless, the sensitivity analysis demonstrates a significant reduction in ejaculate volume in *U. urealyticum-*positive patients when compared with the controls (SMD 0.52 [95% CI: 0.24, 0.81] *p* = 0.0004) and a significant heterogeneity was also observed (I^2^ = 95%; *X*^2^ *p* < 0.00001) (Fig. 2).

#### Seminal fluid pH

**Fig. 3:**
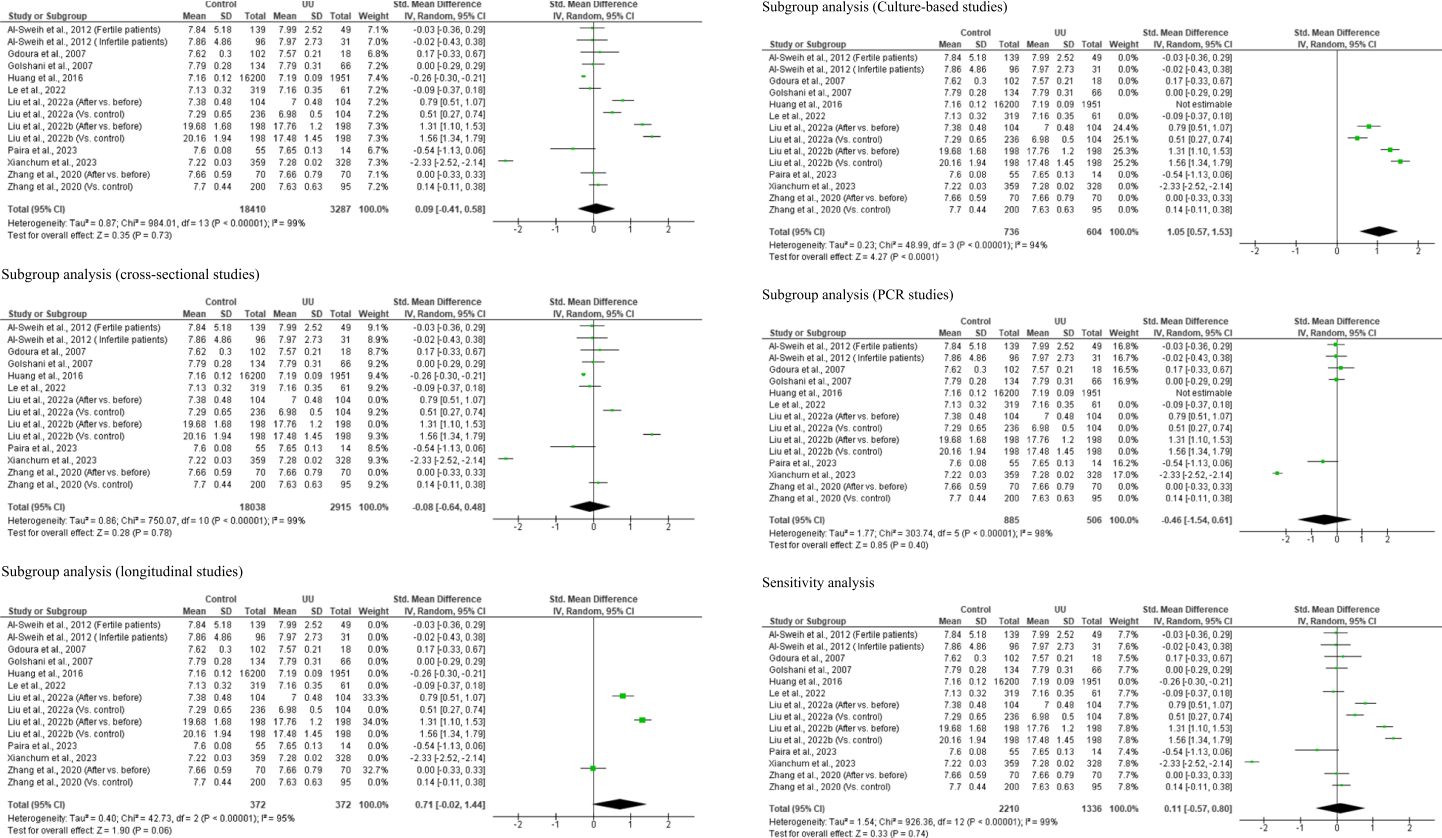
Effect of *ureaplasma urealyticum* on seminal fluid pH. UU: *Ureaplasma urealyticum,* SD: Standard deviation, CI: confidence interval. Meta-analysis was performed using a random-effect model. Data are shown as standardized mean difference and Confidence interval (CI)

A total of 14 studies from 10 articles, consisting of 18, 410 control and 3, 287 *U. urealyticum-positive* subjects, were evaluated for the impact of *U. urealyticum* on seminal fluid pH. It was observed that *U. urealyticum* did not significantly alter seminal fluid pH (SMD 0.09 [95% CI: − 0.41, 0.58] *p* = 0.73) when compared with the controls and a significant heterogeneity was observed (I^2^ = 99%; *X*^2^ *p* < 0.00001). Also, publication bias was observed (S Fig. 2). The subgroup analysis of the cross-sectional studies also revealed that *U. urealyticum* did not significantly alter seminal fluid pH (SMD − 0.08 [95% CI: − 0.64, 0.48] *p* = 0.78) when compared with *U. urealyticum-*negative controls. A significant heterogeneity was observed (I^2^ = 99%; *X*^2^ *p* < 0.00001). More so, the seminal fluid pH did not vary considerably after *U. urealyticum* treatment compared to before treatment (SMD 0.71 [95% CI: − 0.02, 1.44] *p* = 0.06) and there was a significant heterogeneity (I^2^ = 95%; *X*^2^ *p* < 0.00001). Additionally, the subgroup analysis of the culture-based studies showed that *U. urealyticum* significantly reduced seminal fluid pH (SMD 1.05 [95% CI: 0.57, 1.53] *p* < 0.0001) but a significant heterogeneity was also observed (I^2^ = 94%; *X*^2^ *p* < 0.00001), while the subgroup analysis of the PCR-based studies revealed that *U. urealyticum* did not alter ejaculate volume (SMD − 0.46 [95% CI: − 1.54, 0.61] *p* = 0.40) and a significant heterogeneity was also observed (I^2^ = 98%; *X*^2^ *p* < 0.00001). Similarly, the sensitivity analysis demonstrated no significant change in the seminal fluid pH of *U. urealyticum-*positive subjects when compared with the control (SMD 0.11 [95% CI: − 0.57, 0.08] *p* = 0.74) and a significant heterogeneity was observed (I^2^ = 99%; *X*^2^ *p* < 0.00001) (Fig. 3).

#### Sperm concentration

**Fig. 4:**
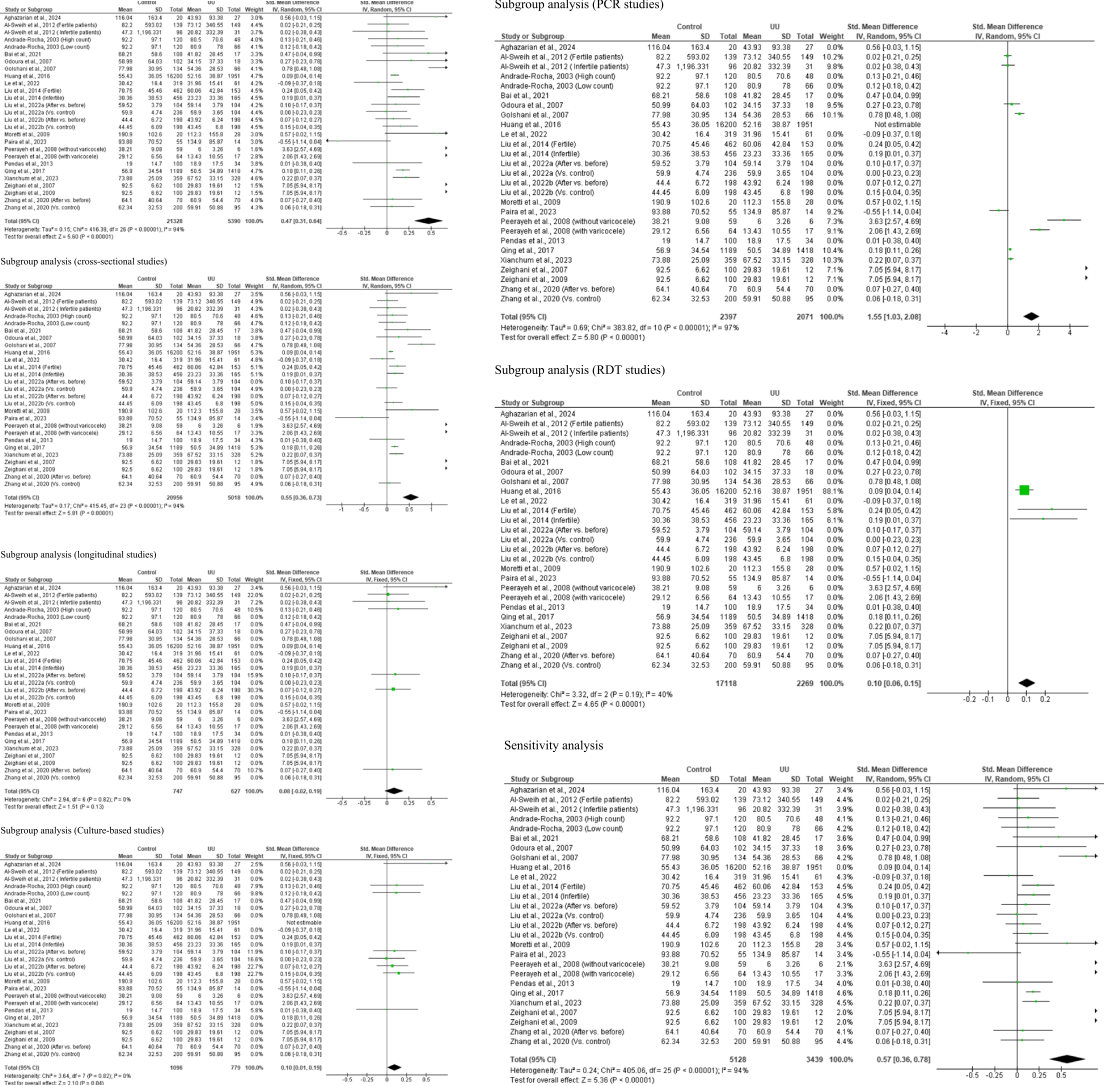
Effect of *ureaplasma urealyticum* on sperm concentration. UU: *Ureaplasma urealyticum,* SD: Standard deviation, CI: confidence interval. Meta-analysis was performed using a random-effect model when *p*-value < 0.1 or I.^2^ > 50%; otherwise, a fixed-model effect is used. Data are shown as standardized mean difference and Confidence interval (CI)

Twenty-seven (27) studies from 20 articles comprising of 21, 328 control and 5, 390 *U. urealyticum-*positive patients were evaluated for the impact of *U. urealyticum* infection on sperm concentration. It was observed that *U. urealyticum* led to a marked reduction in sperm concentration when compared with the control (SMD 0.47 [95% CI: 0.31, 0.64] *p* < 0.00001) when compared with the controls and a significant heterogeneity existed (I^2^ = 94%; *X*^2^ *p* < 0.00001). Publication bias was also observed (S Fig. 3). In addition, a subgroup analysis of the cross-sectional studies revealed that *U. urealyticum-*positive patients had significantly lower sperm concentration when compared with *U. urealyticum-*negative subjects (SMD 0.55 [95% CI: 0.36, 0.73] *p* < 0.00001); however, there was a significant heterogeneity (I^2^ = 94%; *X*^2^ *p* < 0.00001). The subgroup analysis of longitudinal studies showed that the sperm concentration of *U. urealyticum*-infected subjects did not significantly improve after treatment compared to before treatment (SMD 0.08 [95% CI: − 0.02, 0.19] *p* = 0.13) and there was no significant heterogeneity (I^2^ = 0%; *X*^2^ *p* = 0.82). Furthermore, the subgroup analyses of the culture-based studies (SMD 0.10 [95% CI: 0.01, 0.19] *p* = 0.04), PCR studies (SMD 1.55 [95% CI: 1.03, 2.08] *p* < 0.00001), and RDT studies (SMD 0.10 [95% CI: 0.06, 0.15] *p* < 0.00001) revealed that *U. urealyticum* significantly reduced sperm concentration when compared with the control, and there was no significant heterogeneity for the culture-based studies (I^2^ = 0%; *X*^2^ *p* = 0.82) and RDT studies (I^2^ = 40%; *X*^2^ *p* < 0.19), but there was for the PCR studies (I^2^ = 97%; *X*^2^ *p* < 0.00001). Nonetheless, sensitivity analysis demonstrated a significant reduction in sperm concentration in *U. urealyticum-*positive subjects when compared with the controls (SMD 0.57 [95% CI: 0.36, 0.78] *p* < 0.00001); but this showed a significant heterogeneity (I^2^ = 94%; *X*^2^ *p* < 0.00001) (Fig. 4).

#### Sperm total motility

**Fig. 5:**
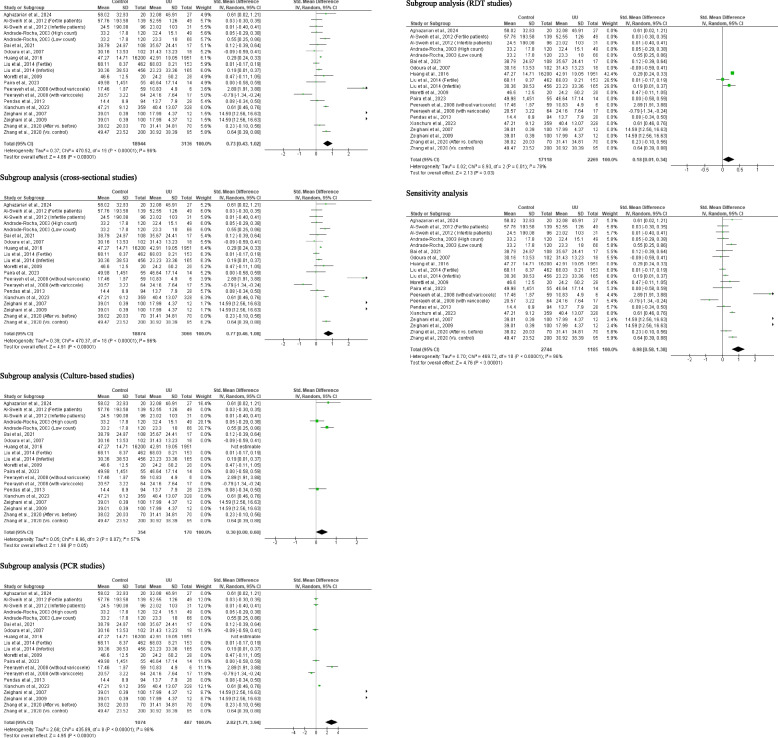
Effect of *ureaplasma urealyticum* on sperm total motility. UU: *Ureaplasma urealyticum,* SD: Standard deviation, CI: confidence interval. Meta-analysis was performed using a random-effect model. Data are shown as standardized mean difference and Confidence interval (CI)

A total of 20 studies from 15 articles that include 18, 944 controls and 3, 136 *U. urealyticum-*infected patients were analyzed for the effect of *U. urealyticum* on sperm total motility. *U. urealyticum* infection significantly reduced sperm total motility when compared with the controls (SMD 0.73 [95% CI: 0.43, 1.02] *p* < 0.00001) and a significant heterogeneity was observed (I^2^ = 96%; *X*^2^ *p* < 0.00001). Publication bias was observed (S Fig. 4). More so, subgroup analysis of the cross-sectional studies revealed that sperm total motility was significantly lower in *U. urealyticum-*positive patients compared with the controls (SMD 0.77 [95% CI: 0.46, 1.08] *p* < 0.00001) but a significant heterogeneity was observed (I^2^ = 96%; *X*^2^ *p* < 0.00001). Additionally, the subgroup analyses of the culture-based studies (SMD 0.30 [95% CI: 0.00, 0.60] *p* = 0.05), PCR studies (SMD 2.82 [95% CI: 1.71, 3.94] *p* < 0.00001), and RDT studies (SMD 0.18 [95% CI: 0.01, 0.03] *p* < 0.00001) demonstrated that *U. urealyticum* significantly reduced sperm total motility when compared with the control, but there was significant heterogeneity (I^2^ = 57%; *X*^2^ *p* = 0.07; I^2^ = 98%; *X*^2^ *p* < 0.00001; I^2^ = 78%; *X*^2^ *p* = 0.01 respectively). Furthermore, the sensitivity analysis showed a significantly lower sperm total motility in *U. urealyticum-*positive patients when compared with the controls (SMD 0.98 [95% CI: 0.58, 1.38] *p* < 0.00001) but a significant heterogeneity was also observed (I^2^ = 96%; *X*^2^ *p* < 0.00001) (Fig. 5).

#### Sperm progressive motility

**Fig. 6:**
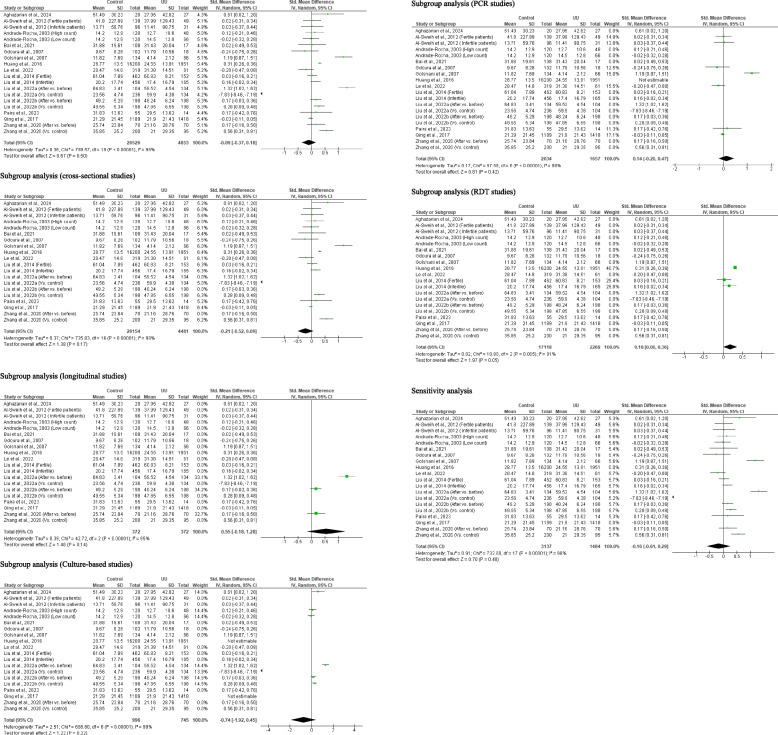
Effect of *ureaplasma urealyticum* on sperm progressive motility. UU: *Ureaplasma urealyticum,* SD: Standard deviation, CI: confidence interval. Meta-analysis was performed using a random-effect model. Data are shown as standardized mean difference and Confidence interval (CI)

Meta-analysis of 20 studies from 14 articles that consisted of 20, 526 controls and 4, 853 *U. urealyticum-*positive subjects revealed that *U. urealyticum* did not significantly alter sperm progressive motility when compared with the controls (SMD − 0.09 [95% CI: − 0.37, 0.18] *p* = 0.50) and a significant heterogeneity was observed (I^2^ = 98%; *X*^2^ *p* < 0.00001). Publication bias was also observed (S Fig. 5). Moreso, the subgroup analysis of the cross-sectional studies revealed no significant difference in sperm progressive motility among *U. urealyticum-*positive subjects when compared with the controls (SMD − 0.21 [95% CI: − 0.52, 0.09] *p* = 0.17) and a significant heterogeneity was observed (I^2^ = 98%; *X*^2^ *p* < 0.00001). Similarly, sperm progressive motility did not significantly change after treatment in *U. urealyticum-*infected subjects when compared with before treatment (SMD 0.55 [95% CI: − 0.18, 1.28] *p* = 0.14) and a significant heterogeneity was observed (I^2^ = 95%; *X*^2^ *p* < 0.00001). More so, subgroup analyses of the culture-based studies (SMD − 0.74 [95% CI: − 1.92, 0.45] *p* = 0.22) and PCR studies (SMD 0.14 [95% CI: − 0.20, 0.47] *p* = 0.42) showed that *U. urealyticum* did not significantly affect sperm progressive motility and there was significant heterogeneity (I^2^ = 99%; *X*^2^ *p* < 0.00001; I^2^ = 90%; *X*^2^ *p* < 0.00001 respectively). However, subgroup analysis of RDT studies showed a reduction of affect sperm progressive motility in *U. urealyticum-*infected individuals when compared with the control (SMD 0.18 [95% CI: 0.00, 0.36] *p* = 0.05) (I^2^ = 81%; *X*^2^ *p* = 0.005). Additionally, sensitivity analysis also revealed that *U. urealyticum* did not significantly alter sperm progressive motility (SMD − 0.16 [95% CI: − 0.61, 0.29] *p* = 0.48) and a significant heterogeneity was observed (I^2^ = 98%; *X*^2^ *p* < 0.00001) (Fig. 6).

#### Total motile sperm count

**Fig. 7:**
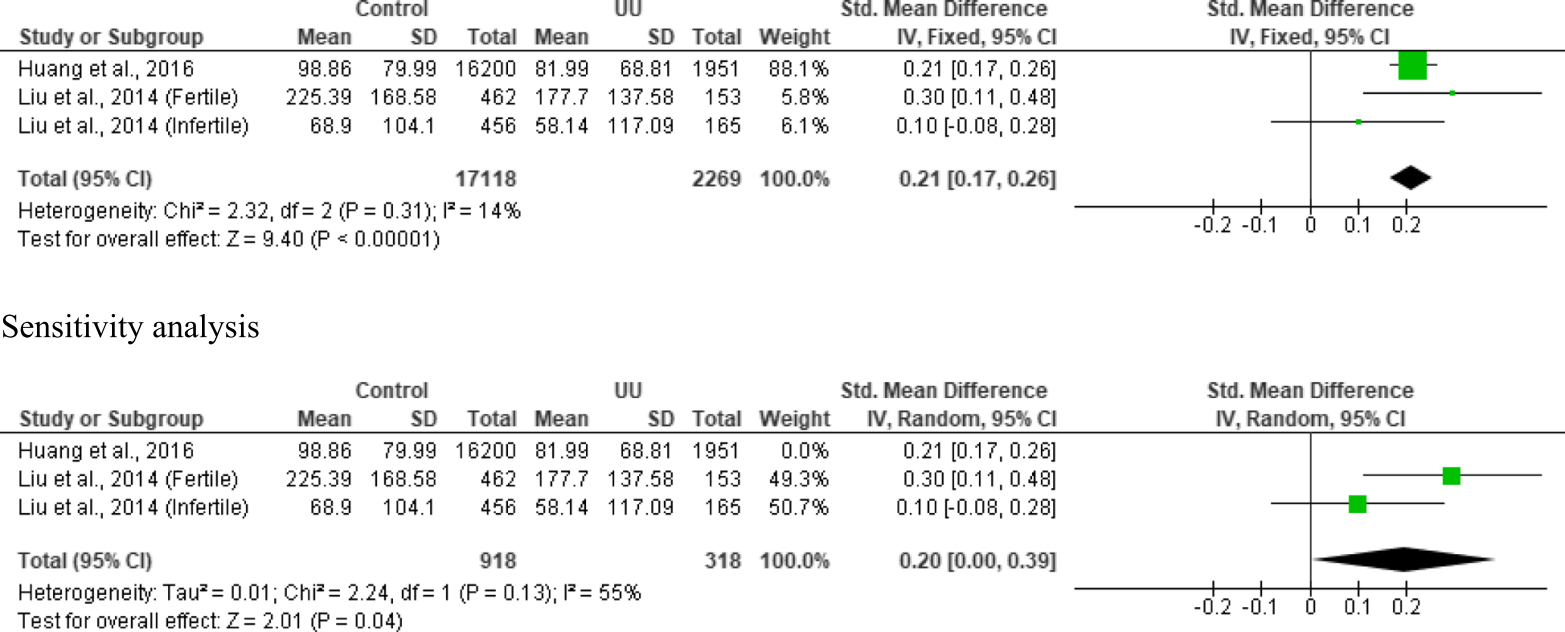
Effect of *ureaplasma urealyticum* on total motile sperm count. UU: *Ureaplasma urealyticum,* SD: Standard deviation, CI: confidence interval. Meta-analysis was performed using a random-effect model when *p*-value < 0.1 or I.^2^ > 50%; otherwise, a fixed-model effect is used. Data are shown as standardized mean difference and Confidence interval (CI)

Three studies from 2 articles, including 17, 118 controls and 2, 269 *U. urealyticum-*positive subjects, were included. *U. urealyticum* significantly reduced total motile sperm count when compared with the control (SMD 0.21 [95% CI: 0.17, 0.26] *p* < 0.00001) and there was no significant heterogeneity (I^2^ = 14%; *X*^2^ *p* = 0.31). There was also no publication bias (S Fig. 6). Sensitivity analysis also demonstrated a significantly reduced total motile sperm count in *U. urealyticum-*positive subjects when compared with the control (SMD 0.20 [95% CI: 0.00, 0.39] *p* = 0.04); however, significant heterogeneity was observed (I^2^ = 55%; *X*^2^ *p* = 0.13) (Fig. 7).

#### Sperm vitality

**Fig. 8:**
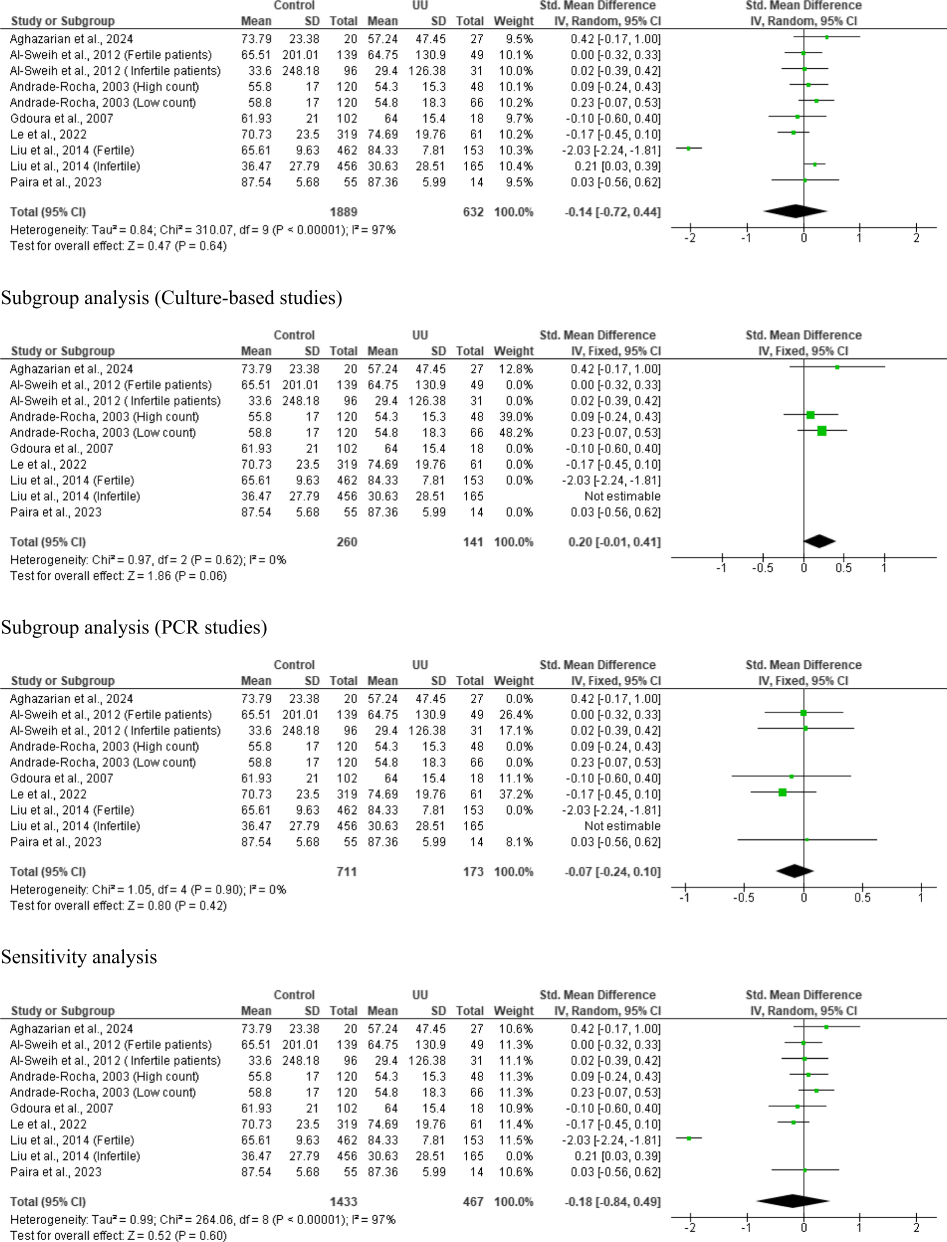
Effect of *ureaplasma urealyticum* on sperm vitality. UU: *Ureaplasma urealyticum,* SD: Standard deviation, CI: confidence interval. Meta-analysis was performed using a random-effect model. Data are shown as standardized mean difference and Confidence interval (CI)

Ten studies from 7 articles consisting of 1, 889 controls and 632 *U. urealyticum-*infected subjects were adjudged eligible for inclusion. *U. urealyticum* infection did not significantly alter sperm vitality when compared with the controls (SMD − 0.14 [95% CI: − 0.72, 0.44] *p* = 0.64) and there was a significant heterogeneity (I^2^ = 97%; *X*^2^ *p* < 0.00001). Publication bias was also observed (S Fig. 7). The subgroup analyses of the culture-based studies (SMD 0.20 [95% CI: − 0.01, 0.41] *p* = 0.06) and PCR studies (SMD − 0.07 [95% CI: − 0.24, 0.10] *p* = 0.42) showed that *U. urealyticum* did not significantly affect sperm vitality and there was no significant heterogeneity (I^2^ = 0%; *X*^2^ *p* = 0.62; I^2^ = 0%; *X*^2^ *p* = 0.90 respectively). More so, sensitivity analysis revealed that *U. urealyticum-*infected subjects had a comparable sperm vitality with *U. urealyticum-*negative subjects SMD − 0.18 [95% CI: − 0.84, 0.49] *p* = 0.60) and there was a significant heterogeneity (I^2^ = 97%; *X*^2^ *p* < 0.00001) (Fig. 8).

#### Sperm normal morphology

**Fig. 9:**
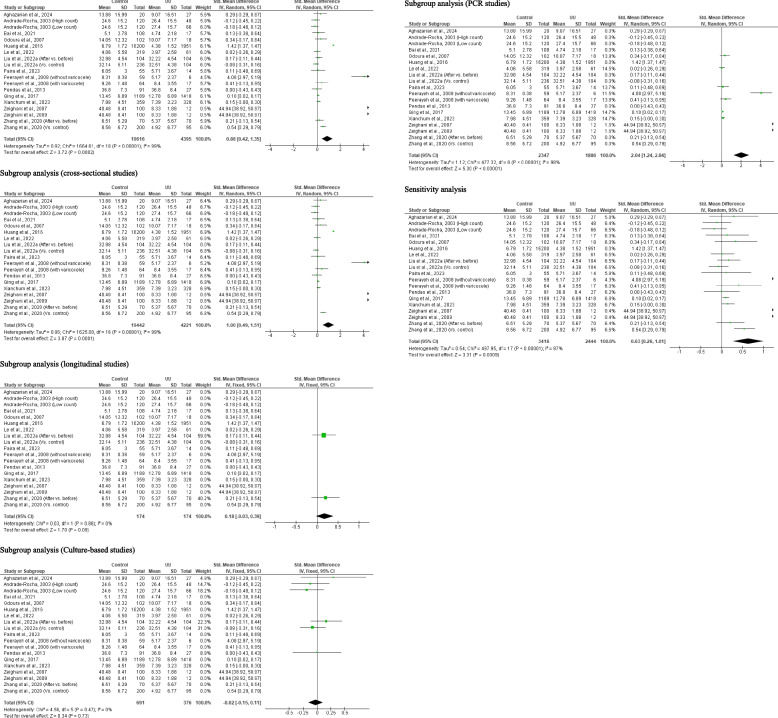
Effect of *ureaplasma urealyticum* on normal sperm morphology. UU: *Ureaplasma urealyticum,* SD: Standard deviation, CI: confidence interval. Meta-analysis was performed using a random-effect model when *p*-value < 0.1 or I.^2^ > 50%; otherwise, a fixed-model effect is used. Data are shown as standardized mean difference and Confidence interval (CI)

A total of 19 studies from 15 published articles consisting of 19, 616 controls and 4, 395 *U. urealyticum*-infected subjects were included to determine the effect of *U. urealyticum* on sperm normal morphology. Subjects who were *U. urealyticum*-infected had a significantly lower sperm normal morphology compared with the control (SMD 0.88 [95% CI: 0.42, 1.35] *p* = 0.0002) and a significant heterogeneity was observed (I^2^ = 99%; *X*^2^ *p* < 0.00001). Publication bias was also observed (S Fig. 8). The subgroup analysis of the cross-sectional studies also revealed a significantly reduced sperm normal morphology in *U. urealyticum*-infected subjects when compared with the controls (SMD 1.00 [95% CI: 0.49, 1.51] *p* = 0.0001) with a significant heterogeneity (I^2^ = 99%; *X*^2^ *p* < 0.00001). However, the subgroup analysis of the longitudinal studies revealed a non-significantly reduced sperm normal morphology in *U. urealyticum*-infected subjects before treatment when compared with after treatment (control) (SMD 0.18 [95% CI: − 0.03, 0.39] *p* = 0.09) with no significant heterogeneity (I^2^ = 0%; *X*^2^ *p* = 0.86). More so, the subgroup analysis of the culture-based studies showed that *U. urealyticum* did not significantly alter sperm normal morphology (SMD − 0.02 [95% CI: − 0.15, 0.11] *p* = 0.73) and a significant heterogeneity was not observed (I^2^ = 0%; *X*^2^ *p* = 0.47), while the subgroup analysis of the PCR-based studies showed that *U. urealyticum* significantly reduced sperm normal morphology (SMD 2.04 [95% CI: 1.24, 2.84] *p* < 0.00001) but a significant heterogeneity was also observed (I^2^ = 98%; *X*^2^ *p* < 0.00001). Notwithstanding, sensitivity analysis demonstrated significantly reduced sperm normal morphology in *U. urealyticum*-infected subjects when compared with the controls (SMD 0.63 [95% CI: 0.26, 1.01] *p* = 0.0009) with a significant heterogeneity (I^2^ = 97%; *X*^2^ *p* < 0.00001) (Fig. 9).

#### Seminal fluid leukocyte count

**Fig. 10:**
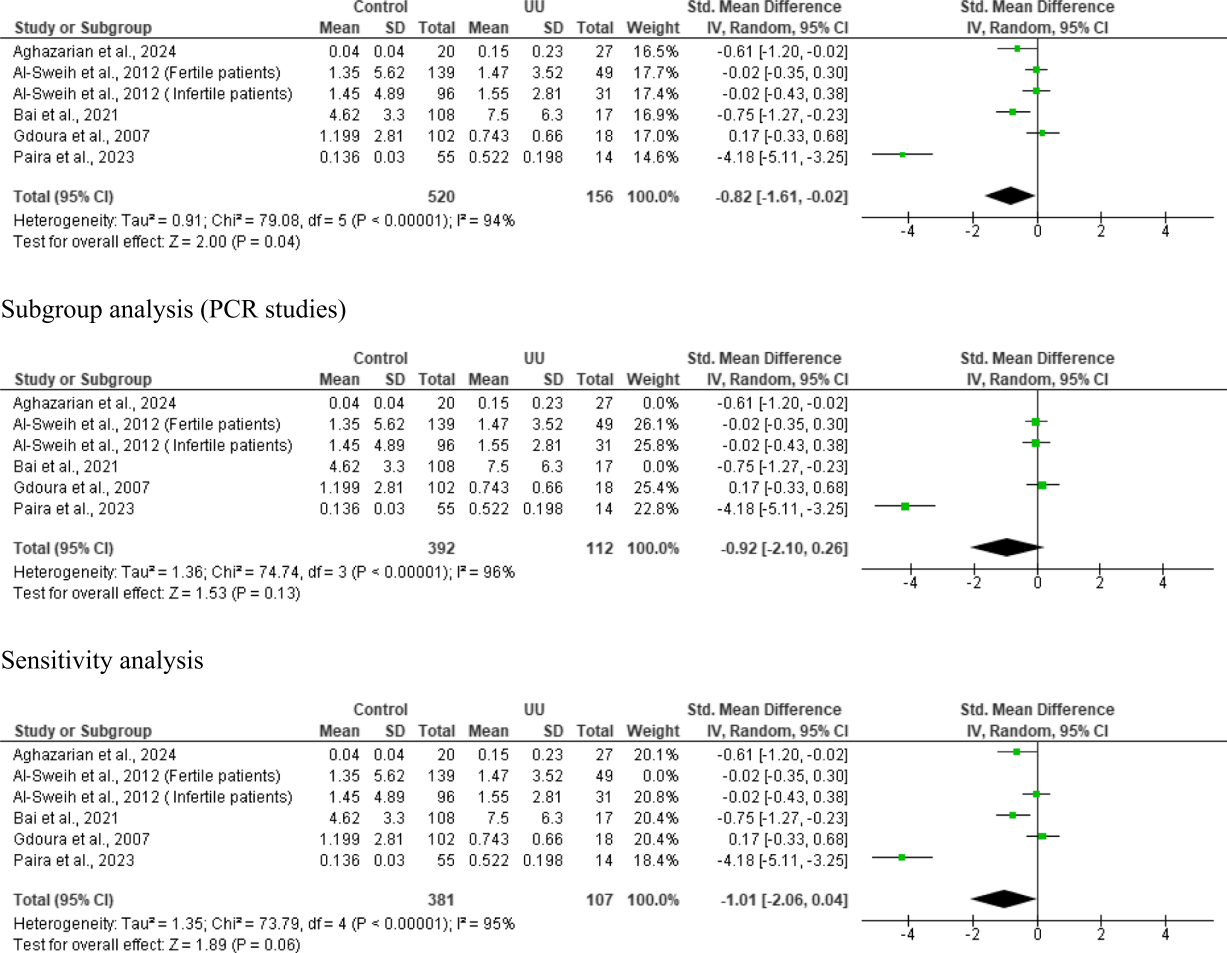
Effect of *ureaplasma urealyticum* on seminal fluid leukocyte count. UU: *Ureaplasma urealyticum,* SD: Standard deviation, CI: confidence interval. Meta-analysis was performed using a random-effect model. Data are shown as standardized mean difference and Confidence interval (CI)

Meta-analysis of six studies from 5 articles consisting of 520 controls and 156 *U. urealyticum*-positive subjects revealed a significantly higher seminal fluid leucocyte count in *U. urealyticum*-positive subjects than *U. urealyticum*-negative controls (SMD − 0.82 [95% CI: − 1.61, − 0.02] *p* = 0.04) and a significant heterogeneity (I^2^ = 94%; *X*^2^ *p* < 0.00001). Publication bias was also observed (S Fig. 9). Subgroup analysis of the PCR-based studies showed that *U. urealyticum* did not significantly alter seminal fluid leucocyte count (SMD − 0.92 [95% CI: − 2.10, 0.26] *p* = 0.13) but a significant heterogeneity was also observed (I^2^ = 96%; *X*^2^ *p* < 0.00001). Sensitivity analysis revealed an insignificantly higher seminal fluid leucocyte count in *U. urealyticum*-positive subjects when compared with the *U. urealyticum*-negative controls (SMD − 1.01 [95% CI: − 2.06, 0.04] *p* = 0.06) and a significant heterogeneity (I^2^ = 95%; *X*^2^ *p* < 0.00001) (Fig. 10).

#### Seminal fluid IL- 6 levels

**Fig. 11:**
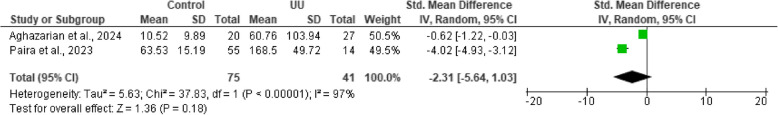
Effect of *ureaplasma urealyticum* on seminal fluid IL- 6. UU: *Ureaplasma urealyticum,* SD: Standard deviation, CI: confidence interval. Meta-analysis was performed using a random-effect model. Data are shown as standardized mean difference and Confidence interval (CI)

Two studies consisting of 75 controls and 41 *U. urealyticum*-positive subjects were included. *U. urealyticum*-positive subjects had an insignificantly higher level of seminal fluid IL- 6 when compared with the control (SMD − 2.31 [95% CI: − 5.64, 1.03] *p* = 0.18) and a significant heterogeneity (I^2^ = 97%; *X*^2^ *p* < 0.00001) (Fig. 11). Publication bias was also observed (S Fig. 10).

### Qualitative analysis

#### Inflammatory markers

Paira et al*.*[11] revealed that subjects who were *U. urealyticum-*positive had a significantly higher levels of IL- 8, tumor necrotic factor-alpha (TNF-α), IL- 1β, and IL- 6 when compared with *U. urealyticum-*negative subjects. They also observed a higher level of peroxidase, *leukocytes*, neutrophils, CD4 + T cells and CD8 + T cells in *U. urealyticum-*positive subjects when compared with their *U. urealyticum-*negative counterparts. In addition, Liu et al*.* [18] demonstrated that seminal fluid elastase was significantly higher in *U. urealyticum-*positive when compared with *U. urealyticum-*negative subjects. They also observed that seminal fluid elastase was significantly higher in *U. urealyticum-*infected subjects before treatment than after treatment. However, Zinzendorf et al. [44]*.* observed an insignificant increase in leucocyte count in *U. urealyticum-*positive subjects when compared with their *U. urealyticum-*negative counterparts.

#### Apoptotic markers

Qing et al. [40] revealed a higher sperm DNA fragmentation in *U. urealyticum-*positive subjects when compared with their *U. urealyticum-*negative counterparts. Also, Moretti et al*.* [37] observed a higher percentage of apoptotic sperm cells in *U. urealyticum-*positive subjects when compared with *U. urealyticum-*negative subjects.

## Discussion

*U. urealyticum* is a common pathogen that is implicated in urogenital infections and may impair male fertility directly via the induction of testicular and sperm toxicity or indirectly via the induction of inflammation and oxidative stress through the upregulation of leukocytes and ROS generation respectively [10]. Despite the reported roles of direct toxicity and oxido-inflammatory injury in *U. urealyticum-*induced male infertility, the precise mechanism underlying the development and progression of low semen quality are yet to be fully understood, and there are ongoing studies exploring the present knowledge dearth. The present meta-analysis is the first, to the best of our knowledge and as at the time of submitting this study for publication, to evaluate the impact of *U. urealyticum* on semen quality and provide possible pathogenetic mechanisms of *U. urealyticum.* This study also performed subgroup analyses for controlled cross-sectional and longitudinal studies.

Conventional semen analysis as well as sperm DNA integrity are useful tools in the diagnosis of male infertility [45]. Notably, this study demonstrated that *U. urealyticum* significantly reduced ejaculate volume, sperm concentration, total sperm motility, total motile sperm count, and normal morphology. This observed decline in semen quality may be due to the direct toxicity induced by *U. urealyticum* on sperm cells [10], leading to alterations in the sperm form, motility, and count. This also shows that *U. urealyticum* may induce male infertility.

Humans have diverse immune response cells in their reproductive systems and these cells release monokines and lymphokines upon activation. These influence tissues outside of the immune system as well as modulate immunological responses locally [46]. The present study revealed that *U. urealyticum* may modulate reproductive immunology by enhancing sperm elastase via the upregulation of seminal fluid leucocyte. Elastase directly damage sperm by inducing phagocytosis and apoptosis by upregulating pro-inflammatory cytokines and ROS [47]. The increased *leukocytes* that explains the rise in elastase also upregulates ROS generation [47]; thus, *U. urealyticum* promotes pro-inflammatory cytokines and ROS surge in the sperm cells by activating the rise in seminal fluid *leukocytes* and elastase. Elastase has also been reported to indirectly cause male infertility by triggering gonadal and epididymal injury through inflammation [48].

This study also observed that *U. urealyticum* promoted sperm DNA fragmentation, which is likely due to its impact of seminal fluid leucocyte. ROS generation may be a cause or consequence of inflammation [49]. *U. urealyticum*-induced upregulation of *leukocytes* promotes ROS generation [47, 48], which in turn damage sperm DNA, resulting in fragmentation that is detrimental to sperm physiological function and overall male fertility. More so, *U. urealyticum* may disrupt the blood-testis barrier [50], compromising the protective environment for germ cells and inducing direct deleterious effect to the germ cells; thus, reducing sperm concentration, total motility, and normal morphology. Furthermore, *U. urealyticum* infection alters seminal fluid composition, reducing the total antioxidant capacity of the sperm cells antioxidants [12], and promoting increased sperm DNA fragmentation. *Ureaplasma urealyticum* fuses with the head of sperm cells, leading to impairment of the swimming movement of the cell and production of metabolites like H_2_O_2_ and OH^−^ which are toxic to the sperm. This alters the sperm morphology and motility, and causes DNA fragmentation and deformity of the sperm cell [14].

Our pooled overall analysis revealed that *U. urealyticum* reduces ejaculate volume, sperm concentration, total sperm motility, and normal morphology, which was consistent with the sensitivity analysis and subgroup analysis of the controlled cross-sectional studies, but a subgroup analysis of the longitudinal study demonstrated only marginal changes in these variables. This may be due to the treatment choice and duration. Despite the convincing data presented in this study, it bears some limitations. First, publication bias was observed in most of the studies (ejaculate volume, seminal fluid pH, sperm concentration, total motility, progressive motility, vitality, normal morphology, and seminal fluid leukocytes and IL- 6 levels). This can lead to overestimation of the effect size and skewed results, making it appear as though the association between *Ureaplasma urealyticum* and sperm quality is stronger than it actually is. Also, the significant heterogeneity noted in some of the analyses influenced the outcome of the present study. It is likely that the different populations studied, diverse diagnostic methods, and different study design are the sources of heterogeneity. More so, prospective interventional studies were included in the meta-analysis, which may influence our findings due to the impact of the treatments. Furthermore, the different diagnostic methods of *U. urealyticum* (PCR, culture or rapid tests) in the primary studies and the duration of recovery after treatment may be a source of bias and influence the study outcome. Nonetheless, this is the first meta-analysis to investigate the impact of *U. urealyticum* on semen quality using 30 studies from 23 published articles. In addition, this study examined the possible pathogenesis of the impact of *U. urealyticum* on semen quality. Moreover, this study conducted a thorough assessment of the quality of evidence, risk of bias and certainty of evidence of the included study to identify the potential source of bias. Furthermore, subgroup analyses of cross-sectional, longitudinal studies (prospective studies), and different diagnostic methods of *U. urealyticum* (PCR, culture or rapid tests) and sensitivity analyses were performed to attenuate the influence of heterogeneity and RoB, publication bias, and strengthen the reliability of our findings.

## Conclusion

There is a significant decline in semen quality, especially ejaculate volume, sperm concentration, total sperm motility, and normal morphology in *U. urealyticum-*infected patients as compared to the controls, which poses a threat to male fertility. The pathogenesis of *U. urealyticum-*induced lowered sperm quality is complex and involves the upregulation of seminal fluid *leukocytes*, elastase, inflammation, and DNA fragmentation. However, further studies are needed to elucidate the mechanisms underlying the association between *U. urealyticum* and semen quality decline, and to develop effective therapies for this condition.

## Supplementary Information

Supplementary Material 1

## Data Availability

Data will be made available on request.

## Abbreviations

CIs: Confidence intervals
CoE: Certainty of evidence
DNA: Deoxyribonucleic acid
GRADE: Grading of Recommendations Assessment, Development and Evaluation
IL: Interleukin
NGU: Non-gonococcal urethritis
OHAT: Office of Health Assessment and Translation
PCR: Polymerase Chain Reactionm
PECOS: Population, Exposure, Comparator/Comparison, Outcomes, and Study design
PRISMA: Preferred Reporting Items for Systematic Reviews and Meta-analyses
QoE: Quality of evidence
RDT: Rapid diagnostic Test
RoB: Risk of bias
ROS: Reactive oxygen species
SMD: Standardized mean difference
TNF-α: Tumor necrotic factor-alpha
