# Supplementary material for: Ureaplasma urealyticum upregulates seminal fluid leukocytes and lowers human semen quality: a systematic review and meta-analysis
Source: Basic Clin Androl. 2025 Apr 17;35:14. doi: 10.1186/s12610-025-00262-5 (PMC12004628; doi:10.1186/s12610-025-00262-5)
Supplement: Supplementary file 1 — Supplementary Material 1 [file 12610_2025_262_MOESM1_ESM.doc]

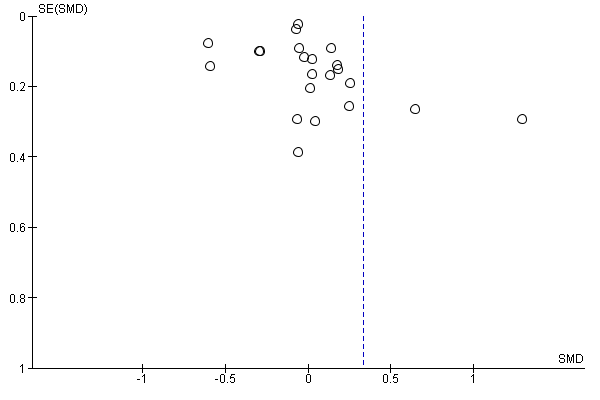


S Fig 1: Publication bias of the studies on the effect of *ureaplasma urealyticum* on human ejaculate volume

SE(SMD): standard error (standardized mean difference)


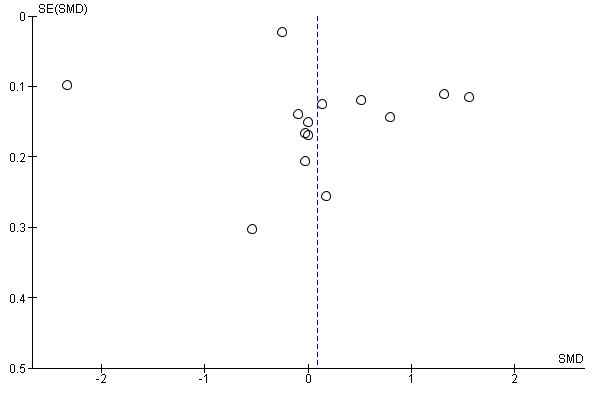


S Fig 2: Publication bias of the studies on the effect of *ureaplasma urealyticum* on seminal fluid pH

SE(SMD): standard error (standardized mean difference)


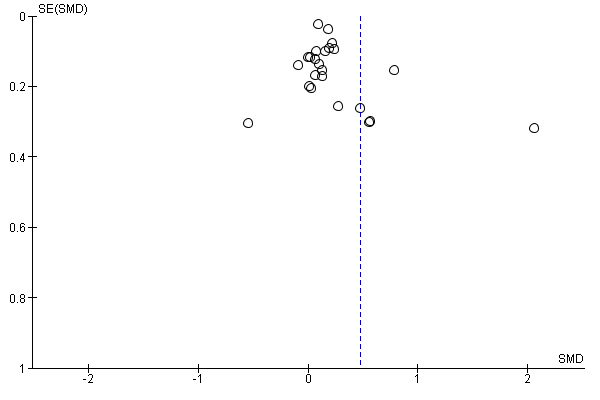


S Fig 3: Publication bias of the studies on the effect of *ureaplasma urealyticum* on sperm concentration

SE(SMD): standard error (standardized mean difference)


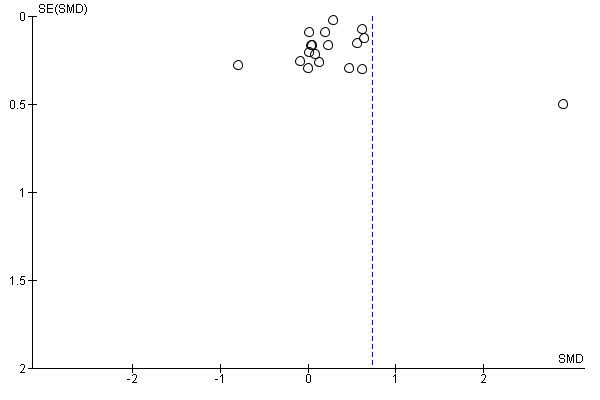


S Fig4: Publication bias of the studies on the effect of *ureaplasma urealyticum* on sperm total motility

SE(SMD): standard error (standardized mean difference)


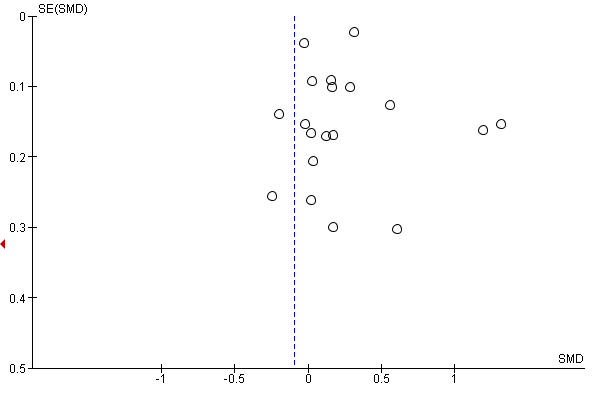


S Fig 5: Publication bias of the studies on the effect of *ureaplasma urealyticum* on sperm progressive motility

SE(SMD): standard error (standardized mean difference)


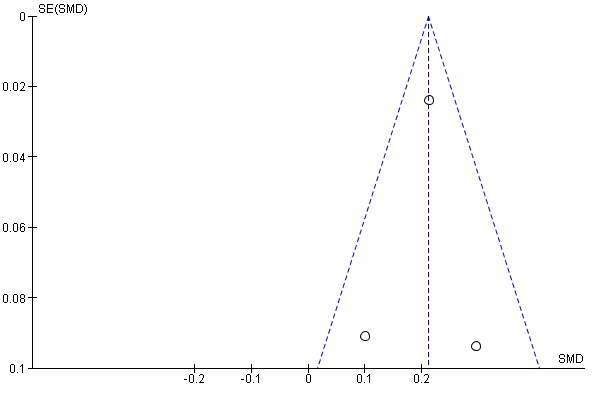


S Fig 6: Publication bias of the studies on the effect of *ureaplasma urealyticum* on total motile sperm count

SE(SMD): standard error (standardized mean difference)


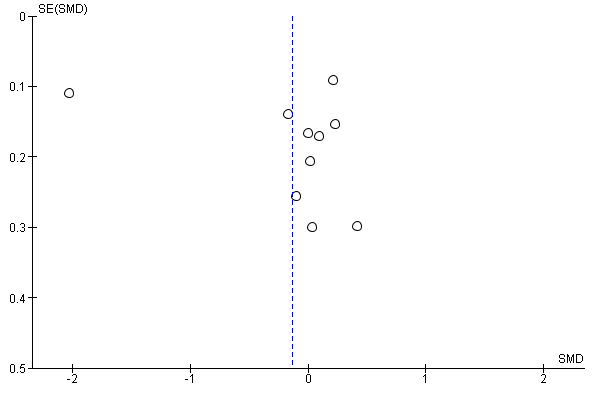


S Fig 7: Publication bias of the studies on the effect of *ureaplasma urealyticum* on sperm vitality

SE(SMD): standard error (standardized mean difference)


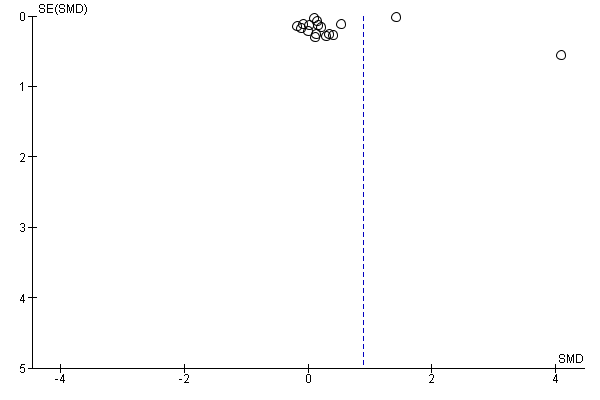


S Fig 8: Publication bias of the studies on the effect of *ureaplasma urealyticum* on normal sperm morphology

SE(SMD): standard error (standardized mean difference)


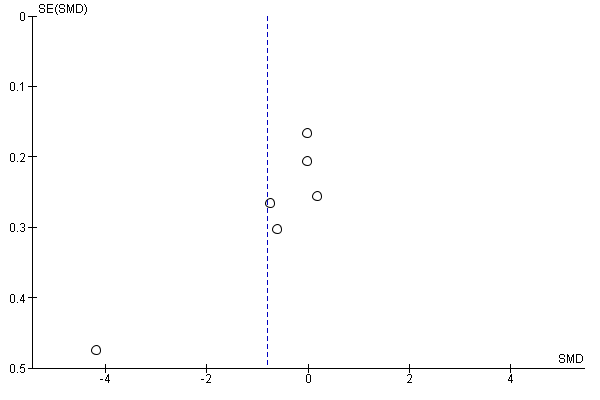


S Fig 9: Publication bias of the studies on the effect of *ureaplasma urealyticum* on seminal fluid leukocyte count

SE(SMD): standard error (standardized mean difference)


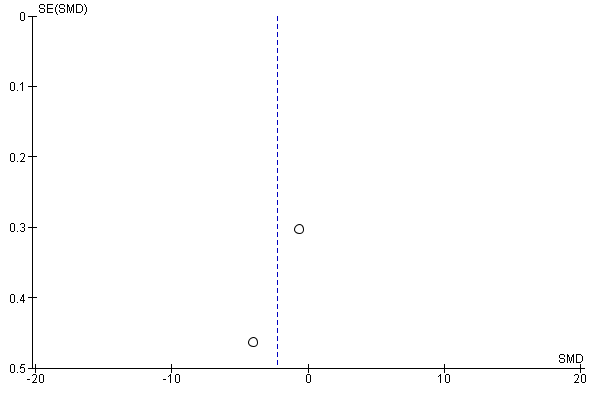


S Fig 10: Publication bias of the studies on the effect of *ureaplasma urealyticum* on seminal fluid IL-6

SE(SMD): standard error (standardized mean difference)
